# Bias of time‐varying exposure effects due to time‐varying covariate measurement strategies

**DOI:** 10.1002/pds.5328

**Published:** 2021-08-01

**Authors:** Bas B. L. Penning de Vries, Rolf H. H. Groenwold

**Affiliations:** ^1^ Department of Clinical Epidemiology Leiden University Medical Center Leiden The Netherlands; ^2^ Department of Biomedical Data Sciences Leiden University Medical Center Leiden The Netherlands

**Keywords:** inverse probability weighting, post‐baseline covariate measurement, simulation, study design, time‐varying confounding

## Abstract

**Purpose:**

In studies of effects of time‐varying drug exposures, adequate adjustment for time‐varying covariates is often necessary to properly control for confounding. However, the granularity of the available covariate data may not be sufficiently fine, for example when covariates are measured for participants only when their exposure levels change.

**Methods:**

To illustrate the impact of choices regarding the frequency of measuring time‐varying covariates, we simulated data for a large target trial and for large observational studies, varying in covariate measurement design. Covariates were measured never, on a fixed‐interval basis, or each time the exposure level switched. For the analysis, it was assumed that covariates remain constant in periods of no measurement. Cumulative survival probabilities for continuous exposure and non‐exposure were estimated using inverse probability weighting to adjust for time‐varying confounding, with special emphasis on the difference between 5‐year event risks.

**Results:**

With monthly covariate measurements, estimates based on observational data coincided with trial‐based estimates, with 5‐year risk differences being zero. Without measurement of baseline or post‐baseline covariates, this risk difference was estimated to be 49% based on the available observational data. With measurements on a fixed‐interval basis only, 5‐year risk differences deviated from the null, to 29% for 6‐monthly measurements, and with magnitude increasing up to 35% as the interval length increased. Risk difference estimates diverged from the null to as low as −18% when covariates were measured depending on exposure level switching.

**Conclusion:**

Our simulations highlight the need for careful consideration of time‐varying covariates in designing studies on time‐varying exposures. We caution against implementing designs with long intervals between measurements. The maximum length required will depend on the rates at which treatments and covariates change, with higher rates requiring shorter measurement intervals.

**Key Points**

- Adequate information on both baseline and time‐varying covariates is important for confounding control in observational studies on the effects of time‐varying drug exposures.
- Using simulated data, it was illustrated that considerable bias might arise when time‐varying covariates are measured on a fixed‐interval bases with long intervals between measurements or when subjects' covariates are assumed to remain constant in periods where their exposure levels remain constant.
- Whether or not data on time‐varying covariates are collected with every exposure level switch, measurement strategies with long intervals between measurements are discouraged.

## 1 INTRODUCTION

In many pharmacoepidemiologic studies, the use of the drugs that are investigated may change over time. In case of such time‐varying exposures, the exposure effect can be defined in different ways. For example, one could contrast initiating drug treatment at a particular point in time (irrespective of whether the use is continued) with not initiating, or continuous drug use with continuous non‐use. While analyses of point interventions (e.g., a single‐dose vaccination) require adjustment for confounding at baseline only, for analyses of a time‐varying exposure, information on time‐varying covariates might be required to mitigate bias due to time‐varying confounding. However, the granularity of the available information about the time‐varying covariates may not be sufficiently fine to adequately control for confounding.

One special case of where this issue may arise is where researchers choose to measure covariates for study subjects only when their exposure levels have changed since the last measurement. If exposure levels do not change, covariate levels are (implicitly) assumed to remain constant, which is an implementation of a method generally known as last‐observation‐carried‐forward (LOCF). The accurateness of the observed covariate data may then depend on the observed exposure history. In studies of antidepressant use and the risk of hip fracture, for example, comorbidities and use of comedication were assessed only at baseline and whenever patients switched exposure level or after every 6 months in the absence of switching.^1^, ^2^

In this paper, we investigate the impact of various covariate measurement designs on the estimation of time‐varying exposure effects in observational studies with time‐varying confounding. We illustrate, by way of simulation, the potential for bias of inverse‐probability‐weighting (IPW) estimators under static designs of fixed‐interval covariate measurement and under dynamic designs with covariates being measured depending on the observed exposure history. IPW estimators are considered as these are increasingly used for estimating causal effects of time‐varying exposures, can accommodate exposure‐covariate feedback,^3^ and readily allow for ‘adjusted’ survival curves to be created.^4^

## 2 METHODS

We first simulated data for a hypothetical study, the ‘target trial’, which if implemented on the theoretical population of interest would readily allow us to identify the exposure effect of interest.^5^ In practice, it is not always possible to implement a target trial, but we use it here as a means to clarify the exposure effect of interest and we simulate from it to give a reference against which to compare results from analyses that are based on simulated data for observational studies. We considered multiple observational studies, each with the same data‐generating mechanism but with different covariate measurement designs to evaluate their impact. Having simulated data, we then estimated the survival curves for the period of 5 years, using a weighting approach (described below) that was designed to keep treatment arms comparable throughout follow‐up in terms of measured covariates. For each of the trial and observational studies, we first generated data on a single sample of *n* = 150 000 individuals, which is sufficiently large to allow us to ignore sampling variability and regard differences between the survival curves as measures of the impact of the measurement designs on the large sample bias of the IPW estimators. The results corresponding to this single simulation run are described in detail below. In Appendix S2, we summarise the results of 5000 independent simulation runs for samples sizes 150 000, 10 000, 1000, 100. R code for this simulation is provided as Appendix S1.

### 2.1 Set‐up

The target trial has the following key design elements: (1) study participants (subjects who satisfy the eligibility criteria) are randomised at a well‐defined baseline time point _*t*0_ to either being issued a drug prescription (*A*_0_ = 1)—say, a prescription for a daily dose of some antidepressant drug for the next one‐month period—or to not being issued the prescription (*A*_0_ = 0) at *t*_0_; (2) participants are then followed over time until the occurrence of an event (e.g., the first hip fracture or death if the subject dies without having sustained a hip fracture during follow‐up) or the administrative study end, whichever comes first; (3) provided event‐free survival is long enough, study participants in the (*A*_0_ = 1)‐group are issued a further prescription after every month since *t*_0_ and those in the (*A*_0_ = 0)‐group do not receive a prescription during follow‐up. For a given subject, we define *A*_*k*_ to be the indicator variable that takes the value of 1 if the subject is on a one‐month prescription on month *k*; *A*_*k*_ = 0 otherwise. We further define *Y* to be the amount of follow‐up time between baseline and the subject's (first) event and let *Y*_*k*_ be that part of _*Y*_ that relates to month _*k*_. We stipulate that study participants are event‐free at the start of the study and that subjects do not get lost to follow‐up before the administrative study end, which we stipulated to be 5 years (or *K* = 60 months) post‐baseline.

The observational studies differ from the target trial in the following ways only: (1) the decision to allocate a subject to *A*_0_ = 1 versus *A*_0_ = 0 is not made by randomisation; (2) the decisions to renew prescriptions for subjects in the (*A*_0_ = 1)‐group or to never issue a prescription throughout the follow‐up period for those in the (*A*_0_ = 0)‐group are not determined by their baseline allocations *A*_0_. Rather, for month *k* = 0,1,…, the decision to set exposure *A*_*k*_ to 0 or 1 is based only on past exposure history (*A*_*j*_: _*j < k*_) and certain binary covariates *L*_*k*_. In this observational setting, subjects can switch at the start of each month between exposure levels ‘being on prescription’ (or ‘exposed’) versus ‘not being on prescription’ (or ‘not exposed’). In variations on this setting, covariate data were measured according to one of the following measurement designs: (1) covariates were not measured at all, thus precluding any adjustment for confounding and effectively forcing us to implement a ‘crude’ estimator; (2) covariates were measured on a monthly basis, which is sufficient for identification of our target quantity; (3) covariates were measured on a 6‐monthly basis starting at baseline; (4) covariates were measured when the respective subject's exposure level switched; (5) covariates were measured with an exposure level switch and at a 6‐monthly basis in the absence of exposure level switching. We also considered variations on designs (3) and (5) where, instead of 6 months, the fixed measurement interval have a length of 2, 3, 9, 12,…, or 60 months. Where design (3) means that measurement times are known before the start of follow‐up, designs (4) and (5) are dynamic in the sense that whether or not a subject's covariate level is measured depends on the subject's time‐varying variables.

### 2.2 Data‐generating mechanism

**FIGURE 1:**
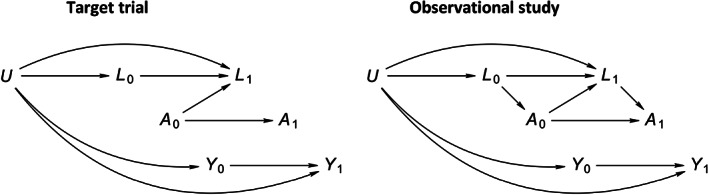
Directed acyclic graphs representing the data‐generating mechanism for the first 2 months of the target trial (left) and observational study (right). Here, *U* represents a unmeasured common cause of the measured covariates *L*_*k−*1_,*L*_*k*_ and outcome variables *Y*_*k−*1_,*Y*_*k*_. The absence of directed paths from exposure variables *A*_*k−*1_,*A*_*k*_ to outcome variables _*Yk−*1_,_*Yk*_ reflects the absence of a causal exposure‐outcome effect

To simulate longitudinal data for a setting with time‐varying confounding we used a variation on the approach described by Havercroft and Didelez^6^ and Young and Tchetgen Tchetgen.^7^ The data‐generating mechanisms for the target trial and observational studies are described in the Appendix A and produce data that are consistent with the directed acyclic graphs (DAGs) of Figure 1. In the trial setting (left panel of Figure 1), the absence of arrows going into the exposure variables reflects the absence of (time‐varying) confounding. In the target trial, post‐baseline exposures are fully determined by the baseline level of exposure, which takes the value of 1 for half of subjects (i.e., exposure status does not change over time). In the observational study, however, approximately 40% of subjects will have switched exposure level by the end of follow‐up in each of the arms that are defined by baseline exposure level.

### 2.3 Defining and estimating the exposure effect

We define the exposure effect of interest as a contrast between continuous exposure (*A*_*j*_ = 1 for *j* = 0,1,…) versus continuous non‐exposure (*A*_*j*_ = 0 for *j* = 0,1,…). In particular, we suppose that the interest lies with a contrast between the 5‐year event‐free survival probabilities that we would observe had everyone received continuous exposure versus continuous non‐exposure; that is, a contrast that is identified in the target trial as

$$\mathrm{Pr}\left(Y\geq 60|A_{0}=1\right)\;\text{versus}\;\mathrm{Pr}\left(Y\geq 60|A_{0}=0\right).$$

As indicated by the absence of a directed path of arrows from the exposure variables to the outcome variables in the DAG for the target trial, the difference between these two survival probabilities is zero.

To account for time‐varying confounding in the observational studies, we implemented IPW by applying a crude (Kaplan–Meier) estimator to an artificial data set where, given a time during follow‐up, a subject received a weight of zero if the subject had experienced an exposure level switch by that time and otherwise a weight equal to the reciprocal of the product of the estimated probabilities of their observed exposure levels until that time given the respective measured exposure and covariate histories. That is, for *a =* 0,1, a subject's weight for month *k* was

$$W_{k}=\prod_{j=0}^{k}\frac{1}{\mathrm{Pr}\left(A_{j}=a|Y\geq j,A_{0}=\ldots =A_{j-1}=a,L_{0},\ldots ,L_{j}\right)}$$

if the subject received exposure level *a* in months 0 through *k* (i.e., *A*_0_ = … = *A*_*k*_ = *a*). Subjects were censored (i.e., received a weight of zero) from the time at which they switched to another exposure level. Apart from the covariate measurement design, the validity of the approach also rests on the correct specification of the model for the conditional treatment probabilities. To ensure correct specification for the reference measurement design (1), we assumed that the exposure _*Ak*_ given survival and past exposure and covariate levels was Bernoulli distributed with mean equal to

$$\mathrm{Pr}\left(A_{k}=1|Y\geq 1,A_{0},\ldots ,A_{k-1},L_{0},\ldots ,L_{k}\right)=\frac{\exp\left[\alpha_{0}+\alpha_{1}I\left(k=0\right)+\alpha_{2}A_{k-1}+\alpha_{3}L_{k}\right]}{1+\exp\left[\alpha_{0}+\alpha_{1}I\left(k=0\right)+\alpha_{2}A_{k-1}+\alpha_{3}L_{k}\right]}$$

for some *α*_0_,*α*_1_,*α*_2_,*α*_3_, which were estimated by a pooled logistic regression under this model. Throughout, variables that were unobserved by measurement design were handled with LOCF.

## 3 RESULTS

**FIGURE 2:**
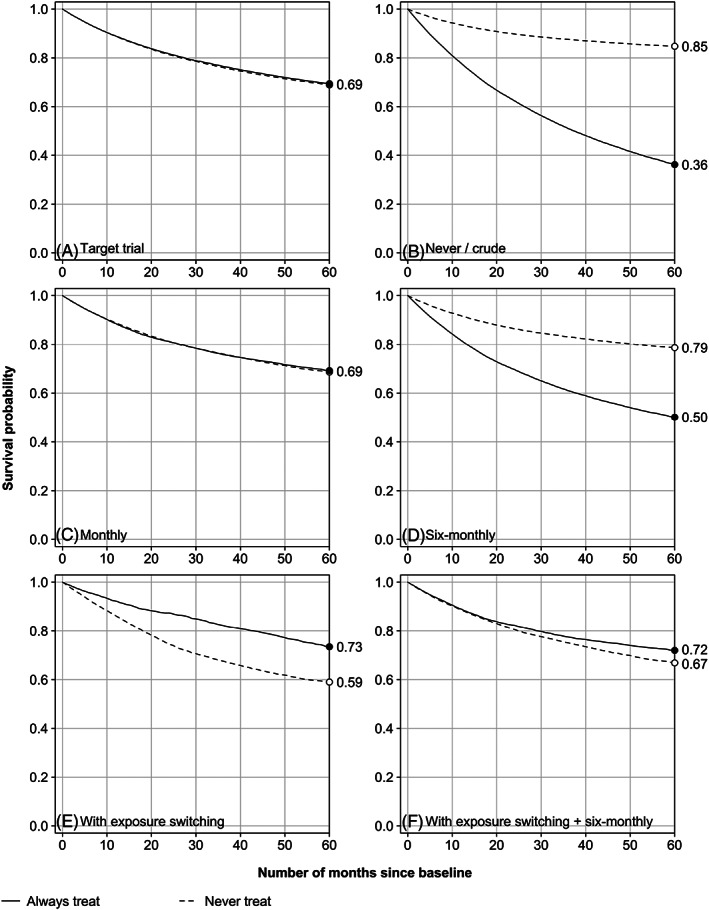
Estimated event‐free survival curves for ‘always treat’ and ‘never treat’ protocols based on target trial (panel A) and observational study (B through F) with varying covariate measurement designs: no covariate measurement B, continuous to monthly covariate measurement C, 6‐monthly covariate measurement D, covariate measurement only with covariate level switching E, and with exposure switching and 6‐monthly in periods without switching F

**FIGURE 3:**
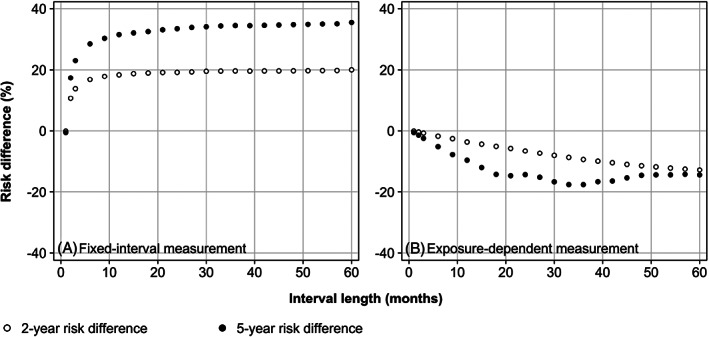
Estimated 2‐ and 5‐year event risk differences comparing ‘always treat’ versus ‘never treat’ protocols. Estimates derive from observational studies with varying covariate measurement designs. Panel A gives the estimates for fixed‐interval measurement; panel B gives the estimates for covariate measurement with exposure switching and with fixed‐length intervals in periods without switching

Figure 2 shows the estimated survival curves for the ‘always treat’ and ‘never treat’ protocols. Consistent with the absence of a directed path from the exposure variables to the outcome variables in the DAGs of Figure 1, the trial‐based estimates of the survival curves overlap (Figure 2, panel A). Where we observed a 5‐year event risk of 31% in both arms of the target trial, in the observational setting, we observed a risk of 64% and 15% in those who do and those who do not receive a treatment prescription at baseline, respectively, giving a risk difference of 49% (panel B). With monthly covariate measurement, IPW resulted in survival curves that virtually coincide with those of the trial (panel C), for which we found a risk difference of zero. Six‐monthly measurements (panel D), however, brought the curves closer to those of the no measurement setting (panel B), that is, in the ‘direction of confounding’. The 5‐year risks with 6‐monthly measurements were estimated to be to 50% and 21%, respectively, giving a risk‐difference of 29%. In Figure 3, panel A, it is shown that the estimated risk differences at 2 and 5 years increase with the interval measurement length, until they reach a plateau of approximately 20% and 35%, respectively. When the interval length was set equal to the maximum follow‐up duration (60 months), only baseline covariates were measured, which resulted in an estimated 5‐year risk difference that was approximately 15% points closer to the target than that of no covariate measurement at all (Figure 2, panel B). When we implemented measurement design (4), the estimated 5‐year risk difference flipped to the other side of the null, −14% (panel E), with 5‐year risks estimated to be 27% and 41% for the ‘always treat’ and ‘never treat’ protocols, respectively. For design (5), we observed a 5‐year risk difference of −5%, somewhere between the results of design (3) and (4) (panel F). With increasingly large measurement intervals within periods of no switching, the estimated 2‐year risk difference steadily decreased to approximately −15% (Figure 3, panel B). The estimated 5‐year risk was also −15% with 60 months between measurements in periods of no switching, equal to the observed risk of design (4), as expected. However, it was lowest, approximately −18%, with an interval length of around 30 months.

The bias estimates of the survival curves and 5‐year risk difference that were derived by averaging across 5000 independent samples of sizes 150 000, 10 000 and 1000 are nearly identical to the corresponding estimates described above (see Supplementary Table and Figures). For sample size 100, however, we observed substantial (small sample) bias for all measurement designs, even in the reference observational setting with full/monthly covariate measurement.

## 4 DISCUSSION

We used simulation to study and illustrate the potential for bias due to measurement design choices in the estimation of the effects of time‐varying exposures. The potential for bias in settings with static or fixed‐interval covariate measurement designs has recently been illustrated already.^8^ We additionally showed that bias might arise in settings where decisions to measure are driven by observed values of the time‐varying exposure.

As expected, in our simulations, fixed‐interval measurement resulted in bias in the direction of confounding, bias that is attributable to residual confounding. Interestingly, we found bias in the opposite direction when we implemented measurement designs where covariates were measured preferentially with exposure level switches. Together with LOCF, these measurement designs introduced a form of differential misclassification, which may result in bias even in the absence of confounding.^9^ Researchers familiar with DAGs might be alerted by the presence of colliders in the DAG that encodes part of the misclassification mechanism. For example, on the DAG of the right panel of Figure 1, the differential misclassification of *L*_1_ can be represented by adding a measured version of *L*_1_ with incoming arrows from *L*_0_, *L*_1_, *A*_0_ and *A*_1_. The measured variable can then be seen to be a collider on the path from *A*_1_ to *Y*_1_ via *L*_1_ and *U*. By conditioning on the collider (and not the unmeasured variable *L*_1_ or *U*), the path is opened, potentially leading to collider‐stratification bias.^3^ In addition to adequate measurement of the time‐varying covariates, the validity of IPW rests on the correct specification of the model for the distribution of the treatment variables given survival and past covariate and exposure levels. It is possible that the biases that we observed are partly due to model misspecification.

We considered a specific and relatively simple setting with a single, binary covariate, no censoring before the administrative study end and an interest in static rather than dynamic treatment strategies. These features are not required for valid inference with IPW.^3^ However, the magnitude and direction of bias in other settings may differ from those observed in the current study. We stress that the bias that was observed in our simulation does not depend critically on the choice of IPW as a means to control for time‐varying confounding. The choices regarding the frequency of covariate measurements will likely also affect other methods, including the commonly applied Cox’ regression analysis with time‐varying covariates. The extent to which such choices impact a particular study are obviously context‐specific. For example, it will likely depend on the rate at which subjects cross over between treatment arms as well as on the extent to which covariates are subject to change over time.

In conclusion, our simulations highlight the need for adequate measurement of time‐varying covariates in observational studies on the effects of time‐varying exposures. Researches should consider differential covariate misclassification as a possible source of bias when designing covariate measurement strategies. Whether or not covariates are measured with every exposure level switch, we caution against implementing measurement designs with long intervals between measurements, particularly when the impact of the design choices are poorly understood. The maximum interval length that is sufficient to yield negligible bias will depend on the rates at which treatments and covariates can change,^8^ with higher rates requiring shorter measurement intervals.

## CONFLICT OF INTEREST

The authors declare no conflict of interest.

## ETHICS STATEMENT

No ethics committee approval was required for this study.

## Supporting information


**Appendix S1.** Supporting Information.

**Appendix S2.** Supporting Information.

## APPENDIX A.

### Data‐generating mechanism

For convenience of notation, define *L*_*k*_ = *A*_*k*_ = 0 if *k* < 0. Also, let *I* and expit denote the indicator and inverse logit function, respectively, so that expit[*x*] = exp[*x*]*/*(1 + exp[*x*]). For all 150 000 study participants, we generated data using independent runs of the following algorithm: (1) generate *U* from a uniform distribution over the interval [0,1], initialise *Y* = 0 and set *k* = 0; (2) if *Y ≥ k*, draw *L*_*k*_ from the Bernoulli distribution with parameter expit[*−*4.25 + 0.25*I*(*k* = 0) +6 *U* + 0.5*A*_*k−*1_ + *L*_*k−*1_] and draw *A*_*k*_ from the Bernoulli distribution with parameter *p*_*k*_; (3) draw *T* from the exponential distribution with rate exp[*−*9.5 + 7 *U*] and increment *Y* with *Y*_*k*_: = min{*T*,1}; (4) if *k* < *K*, increment *k* with 1 and return to (2); stop otherwise. For the observational study, we defined *p*_*k*_ = expit[*−*7 + 4*I*(*k* = 0) + 10*A*_*k−*1_ + 4*L*_*k*_], whereas for the trial we defined *p*_*k*_ = 0.5*I*(*k* = 0) + *A*_*k−*1_ *I*(*k >* 0).
