# Supplementary material for: Bias of time‐varying exposure effects due to time‐varying covariate measurement strategies
Source: Pharmacoepidemiol Drug Saf. 2021 Aug 1;31(1):22–7. doi: 10.1002/pds.5328 (PMC9292390; doi:10.1002/pds.5328)
Supplement: Supplementary file 2 — Appendix S2. Supporting Information. [file PDS-31-22-s001.pdf]

Supplementary material to 'Bias of time-varying exposure effects due to time-varying covariate measurement strategies'

Supplementary Table: Summary of estimated 5-year (always-versus-never-exposed) risk differences over 5000 simulation runs for sample sizes 150 000, 10 000, 1000, and 100.

Study/ measurement design†	Mean estimate (95% CI)‡	Empirical variance	Mean squared error
	Sample size: 150 000		
A: Target trial	-0.000 (-0.000, 0.000)	0.000	0.000
B: Observational study 1	0.485 (0.485, 0.485)	0.000	0.235
C: Observational study 2	-0.000 (-0.000, 0.000)	0.000	0.000
D: Observational study 3	0.286 (0.286, 0.286)	0.000	0.082
E: Observational study 4	-0.134 (-0.134, -0.134)	0.000	0.018
F: Observational study 5	-0.044 (-0.044, -0.043)	0.000	0.002
	Sample size: 10 000		
A: Target trial	0.000 (-0.000, 0.000)	0.000	0.000
B: Observational study 1	0.485 (0.485, 0.486)	0.000	0.236
C: Observational study 2	0.000 (-0.000, 0.001)	0.001	0.001
D: Observational study 3	0.286 (0.286, 0.287)	0.000	0.082
E: Observational study 4	-0.135 (-0.136, -0.134)	0.002	0.021
F: Observational study 5	-0.043 (-0.044, -0.042)	0.001	0.003
	Sample size: 1000		
A: Target trial	0.000 (-0.001, 0.001)	0.001	0.001
B: Observational study 1	0.486 (0.485, 0.487)	0.002	0.238
C: Observational study 2	0.014 (0.012, 0.016)	0.007	0.007
D: Observational study 3	0.291 (0.289, 0.292)	0.003	0.087
E: Observational study 4	-0.132 (-0.136, -0.128)	0.026	0.044
F: Observational study 5	-0.028 (-0.031, -0.025)	0.009	0.009
	Sample size: 100		
A: Target trial	-0.000 (-0.003, 0.003)	0.009	0.009
B: Observational study 1	0.484 (0.481, 0.488)	0.016	0.251
C: Observational study 2	0.117 (0.110, 0.123)	0.060	0.073
D: Observational study 3	0.320 (0.315, 0.324)	0.026	0.129
E: Observational study 4	-0.004 (-0.014, 0.007)	0.154	0.154
F: Observational study 5	0.091 (0.084, 0.099)	0.072	0.080

†The target trial and observational studies are described in the main text. Observational studies 1 through 5 differ in covariate measurement design: in observational study 1 (B), covariates were never measured; in study 2 (C), covariates were measured on a monthly basis; in study 3 (D), covariates were measured on a six-monthly basis starting at baseline; in study 4 (E), covariates were measured when the respective subject's exposure level switched; in study 5 (F), covariates were measured with an exposure level switch and at a six-monthly basis in the absence of exposure level switching. ‡95% CI refers to the pointwise 95% confidence interval $\hat{\mu} \pm 1.96\sqrt{\hat{\sigma}^2/5000}$, where $\hat{\mu}$ denotes the mean estimated risk difference and $\hat{\sigma}^2$ its empirical variance, i.e., the sample variance of the sample of 5000 estimates.

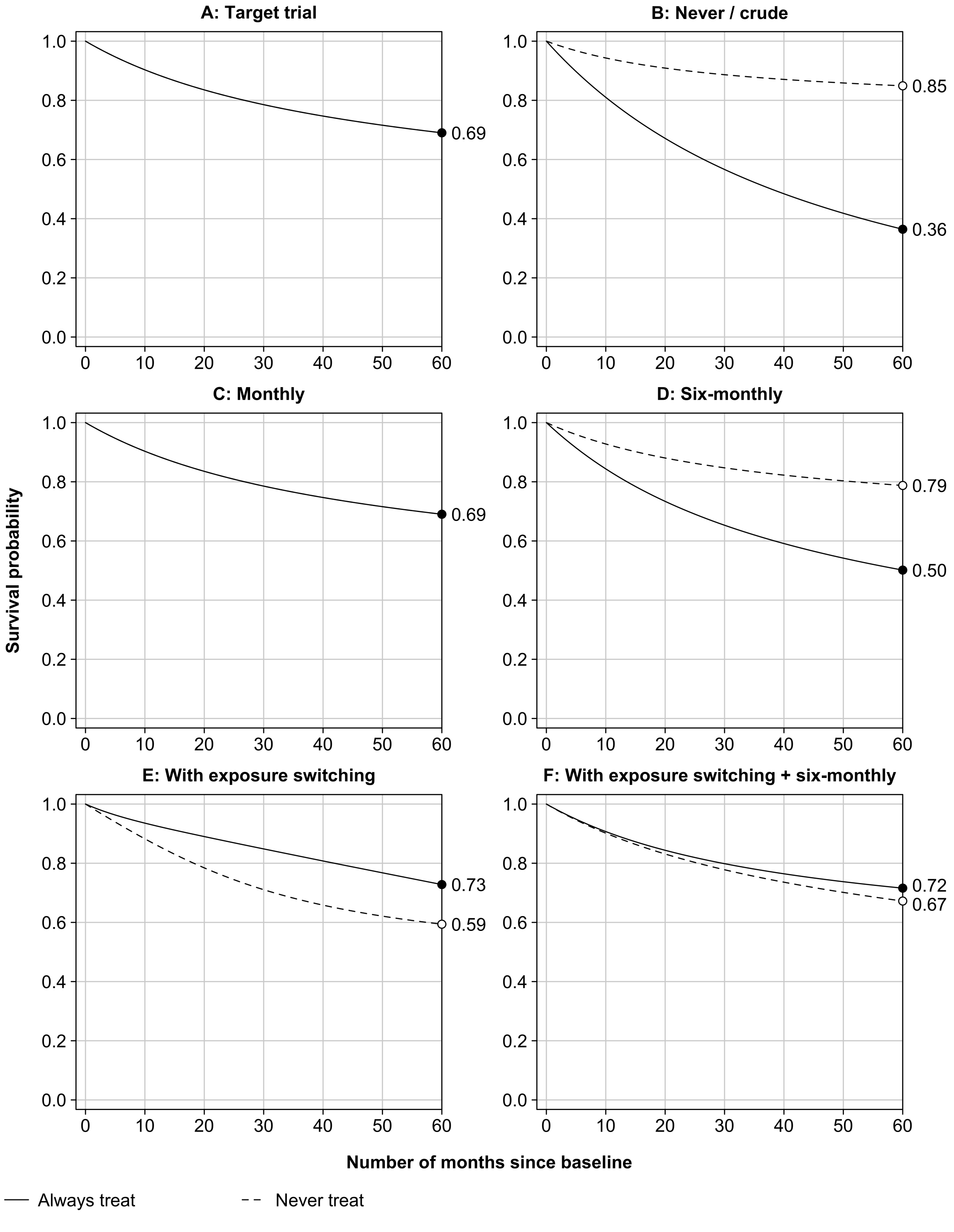

Supplementary Figure 1: Mean estimated event-free survival probabilities across 5000 samples of size 150 000 based on target trial (panel A) and observational study (B through F) with varying covariate measurement designs: no covariate measurement (B), continuous to monthly covariate measurement (C), six-monthly covariate measurement (D), covariate measurement only with covariate level switching (E), and with exposure switching and six-monthly in periods without switching (F).

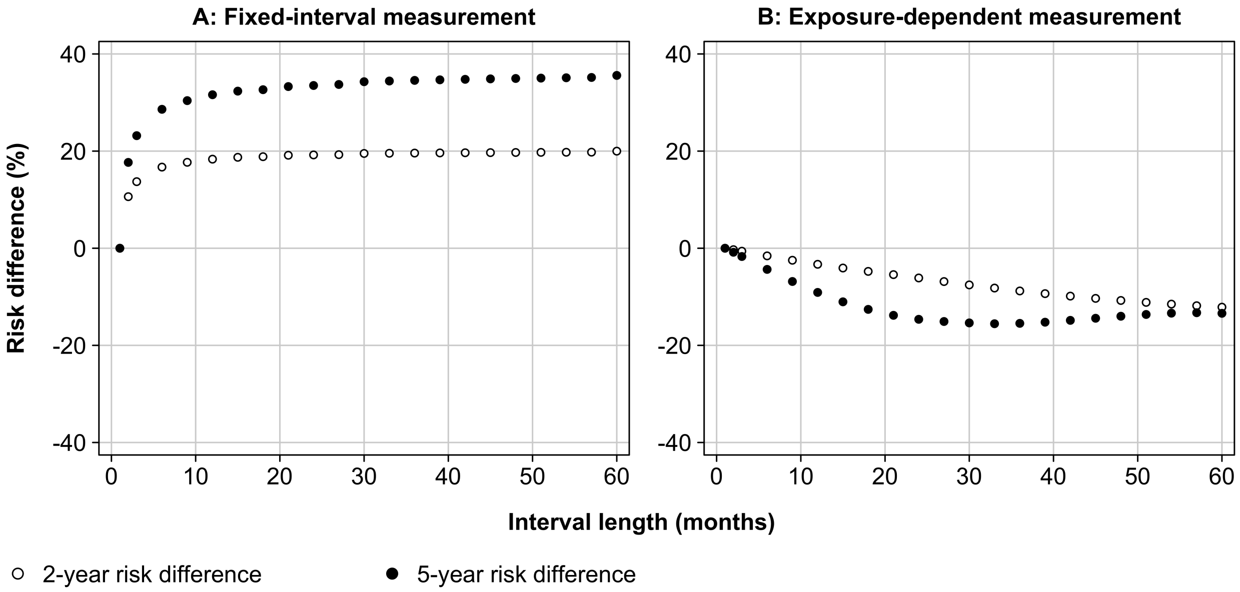

Supplementary Figure 2: Mean estimated two- and five-year event risk differences across 5000 samples of size 150 000. Estimates derive from observational studies with varying covariate measurement designs. Panel A gives the estimates for fixed-interval measurement; panel B gives the estimates for covariate measurement with exposure switching and with fixed-length intervals in periods without switching.

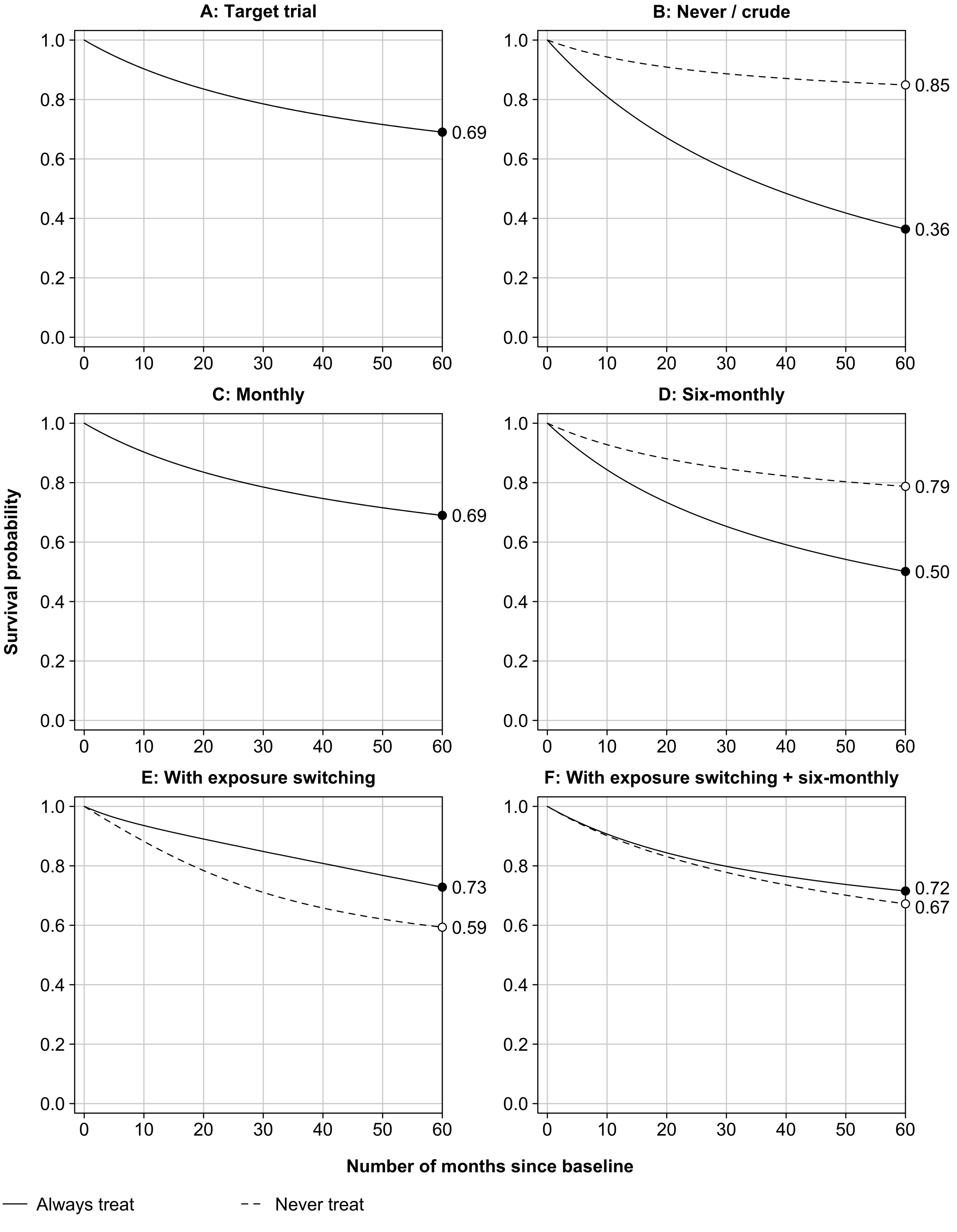

Supplementary Figure 3: Mean estimated event-free survival probabilities across 5000 samples of size 10 000 based on target trial (panel A) and observational study (B through F) with varying covariate measurement designs: no covariate measurement (B), continuous to monthly covariate measurement (C), six-monthly covariate measurement (D), covariate measurement only with covariate level switching (E), and with exposure switching and six-monthly in periods without switching (F).

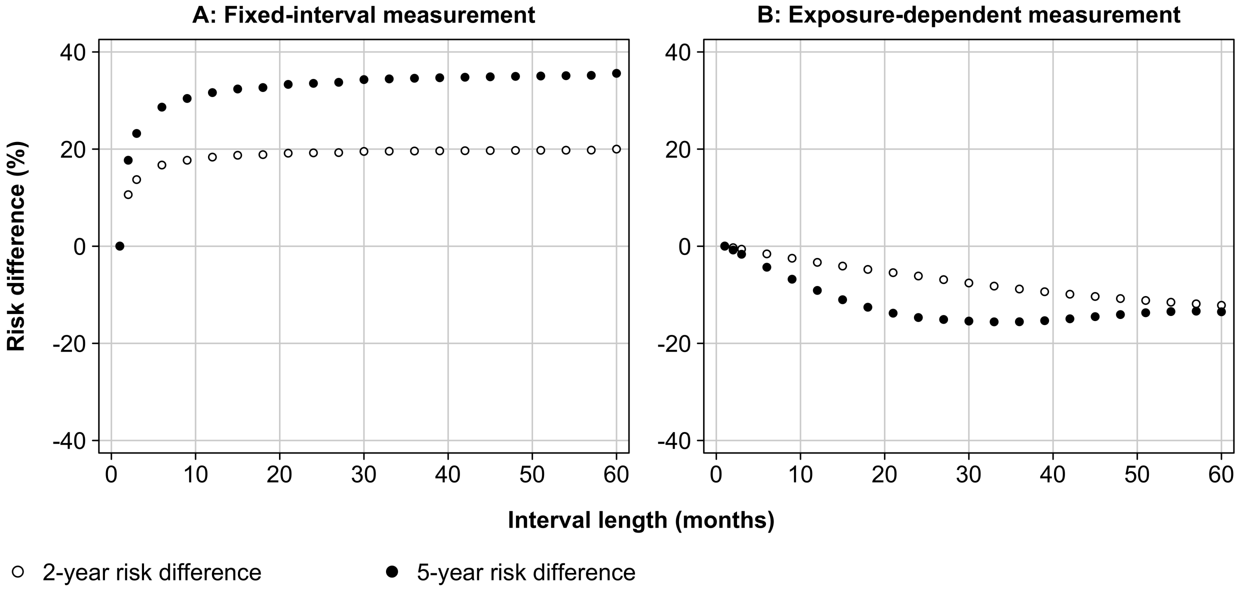

Supplementary Figure 4: Mean estimated two- and five-year event risk differences across 5000 samples of size 10 000. Estimates derive from observational studies with varying covariate measurement designs. Panel A gives the estimates for fixed-interval measurement; panel B gives the estimates for covariate measurement with exposure switching and with fixed-length intervals in periods without switching.

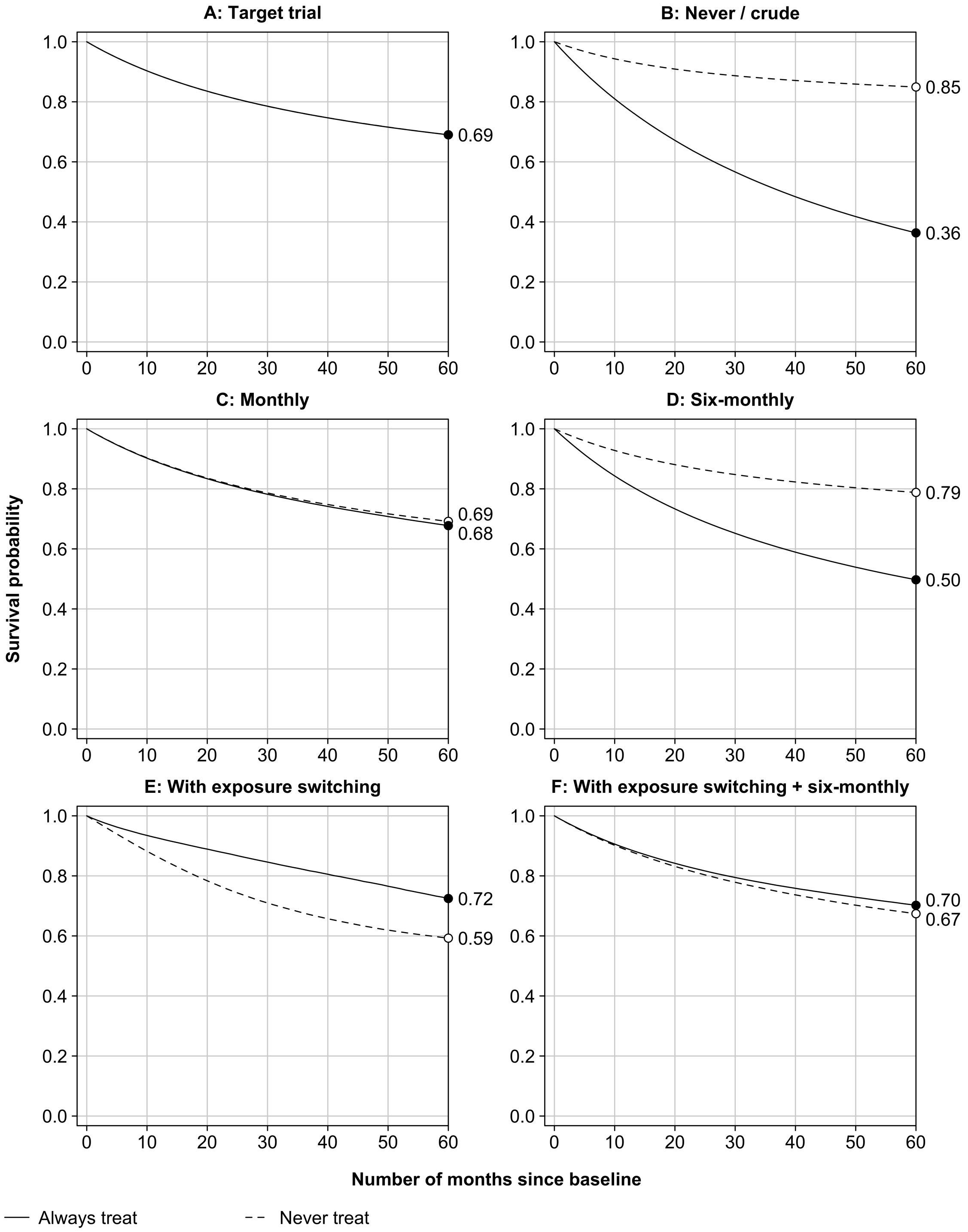

Supplementary Figure 5: Mean estimated event-free survival probabilities across 5000 samples of size 1000 based on target trial (panel A) and observational study (B through F) with varying covariate measurement designs: no covariate measurement (B), continuous to monthly covariate measurement (C), six-monthly covariate measurement (D), covariate measurement only with covariate level switching (E), and with exposure switching and six-monthly in periods without switching (F).

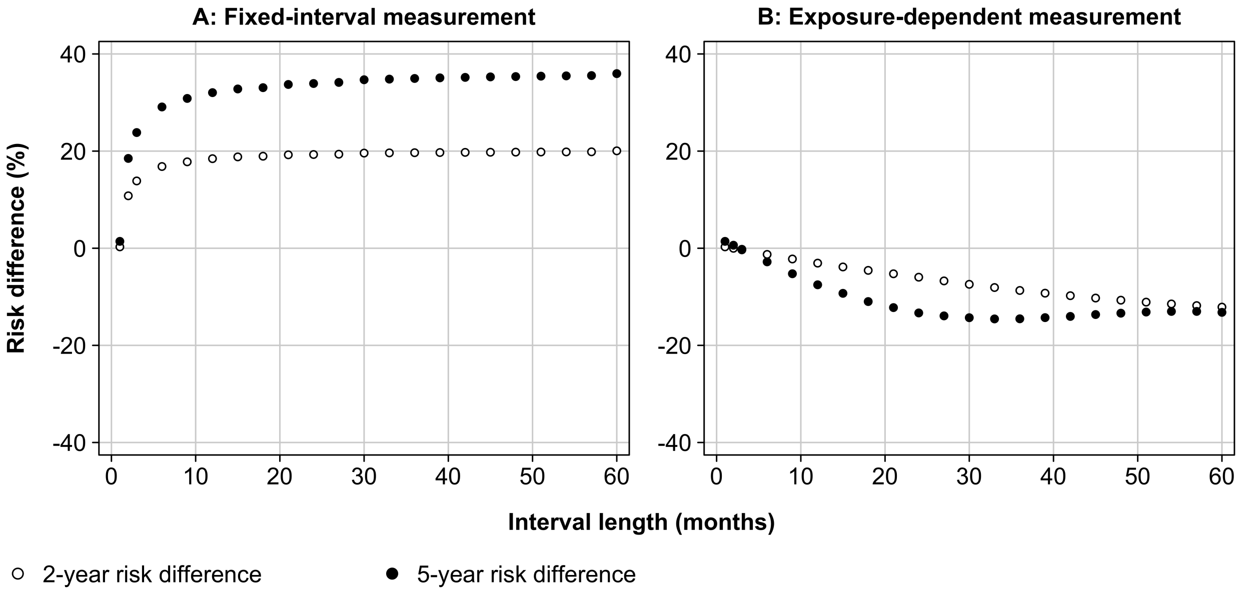

Supplementary Figure 6: Mean estimated two- and five-year event risk differences across 5000 samples of size 1000. Estimates derive from observational studies with varying covariate measurement designs. Panel A gives the estimates for fixed-interval measurement; panel B gives the estimates for covariate measurement with exposure switching and with fixed-length intervals in periods without switching.

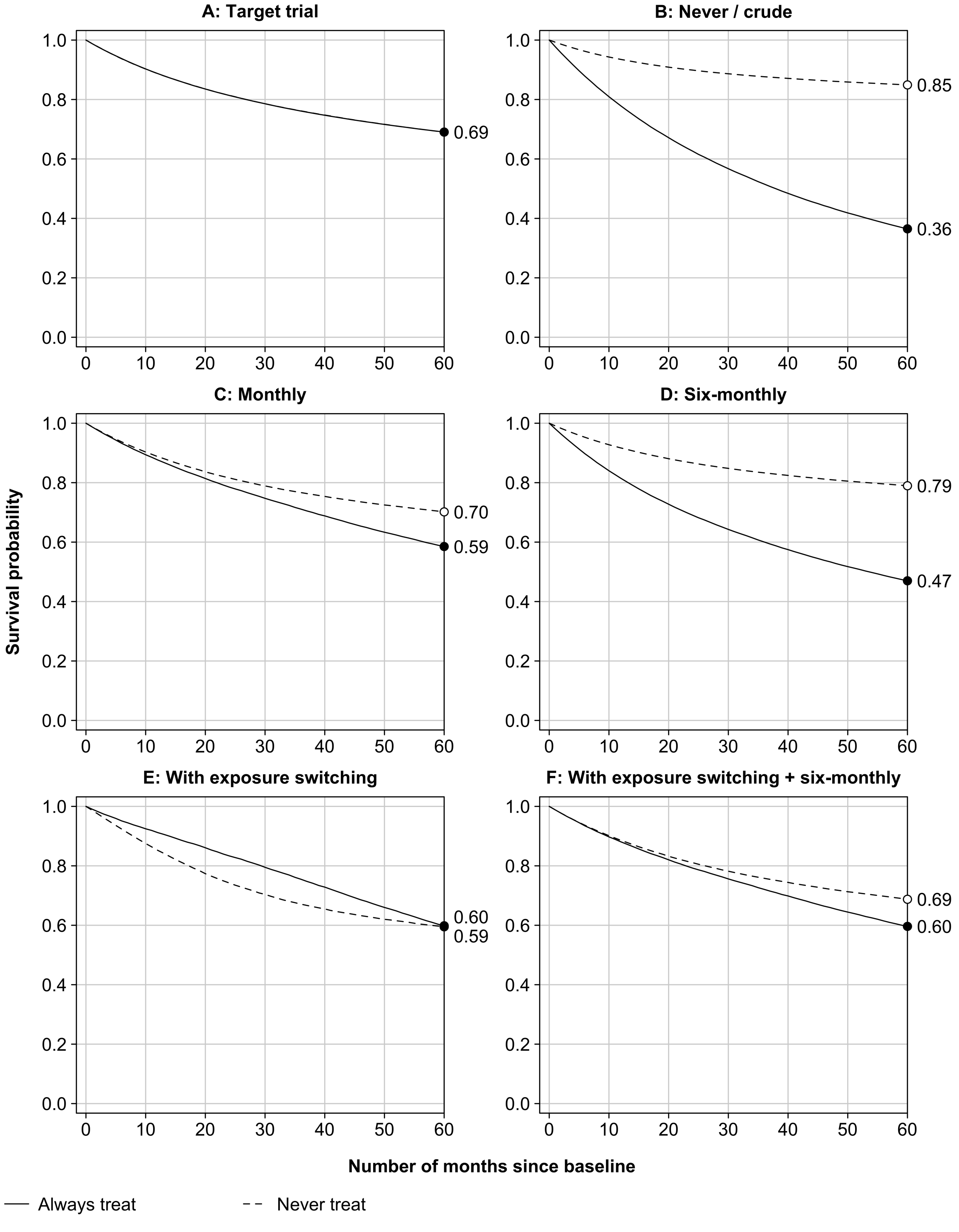

Supplementary Figure 7: Mean estimated event-free survival probabilities across 5000 samples of size 100 based on target trial (panel A) and observational study (B through F) with varying covariate measurement designs: no covariate measurement (B), continuous to monthly covariate measurement (C), six-monthly covariate measurement (D), covariate measurement only with covariate level switching (E), and with exposure switching and six-monthly in periods without switching (F).

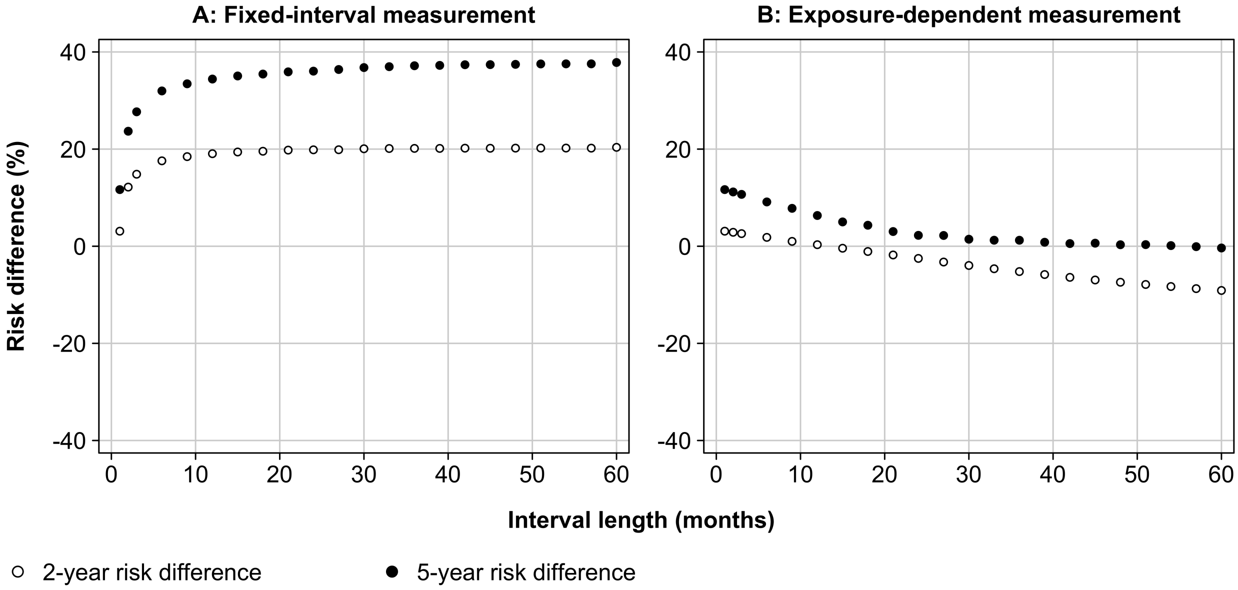

Supplementary Figure 8: Mean estimated two- and five-year event risk differences across 5000 samples of size 100. Estimates derive from observational studies with varying covariate measurement designs. Panel A gives the estimates for fixed-interval measurement; panel B gives the estimates for covariate measurement with exposure switching and with fixed-length intervals in periods without switching.
